# Impact of electrolyte-rich dialysate during continuous renal replacement therapy on serum phosphate and potassium in ICU patients

**DOI:** 10.1371/journal.pone.0238867

**Published:** 2020-09-11

**Authors:** AJin Cho, Young-Ki Lee, Hayne Cho Park

**Affiliations:** Department of Internal Medicine, Kangnam Sacred Heart Hospital, Hallym University College of Medicine, Seoul, Republic of Korea; International University of Health and Welfare, School of Medicine, JAPAN

## Abstract

**Background:**

Hypophosphatemia and hypokalemia occur frequently during continuous renal replacement therapy (CRRT). We evaluated serum phosphate and potassium levels in patients administered three different types of dialysis solution.

**Methods:**

The study population consisted of 324 intensive care unit patients who underwent CRRT between January 2015 and December 2018. Patients were divided into three groups: group 1 (n = 105) received Hemosol B0 (no potassium or phosphate); group 2 (n = 78) received Hemosol B0 and potassium-containing solution (MultiBic); and group 3 (n = 141) received phosphate- and potassium-containing solution (Phoxilium), Hemosol B2, Prismasol 2, and Prismasol 4. A different protocol was followed in each group.

**Results:**

The incidence rate of hypophosphatemia was 55% lower in group 3 compared to group 1 (incidence rate ratio (IRR) 0.45, 95% confidence interval (CI): 0.33 to 0.61) and 61% lower compared to group 2 (IRR 0.39, 95% CI: 0.29 to 0.53). Group 3 also had a 50% lower incidence rate of hypokalemia compared to group 1 (IRR 0.50, 95% CI: 0.29 to 0.88). The negative slope in phosphate level in group 3 was greater than that in group 1 (ß = 0.19, 95% CI: 0.02 to 0.37, p = 0.032), while the negative slope in the potassium level was greater in group 2 than in group 1(ß = 0.10, 95% CI: 0.03 to 0.17, p = 0.008). Additional intravenous calcium was not used in any case, and most cases of acid-base disturbances were well controlled.

**Conclusions:**

The use of phosphate- and potassium-containing with a proper CRRT protocol prevented decreases in serum phosphate and potassium levels, thus also preventing hypophosphatemia and hypokalemia, and additional replacement during CRRT.

## Introduction

Continuous renal replacement therapy (CRRT) has been widely applied in the intensive care unit (ICU) because it is better tolerated by hemodynamically unstable patients [1–3]. However, the increased solute clearance associated with CRRT may lead to undesirable amino acid and electrolyte losses. Indeed, continuous therapy, if monitored inappropriately, can cause several types of electrolyte imbalance. Previous studies reported that 50–65% of patients develop hypophosphatemia, while up to 20% develop hypokalemia, during CRRT [4–6]. Severe hypophosphatemia was reported to be associated with increased ICU mortality [7, 8]. Symptoms of hypophosphatemia, including ventilatory muscle weakness, prolonged ventilator dependence, seizure, cardiac arrhythmia, rhabdomyolysis, and hemolysis, are usually seen in patients with moderate or severe hypophosphatemia [9–13]. Serum potassium level also has a significant impact on ICU patients, with hypokalemia known to cause ventricular arrhythmias and to be linked to increased mortality [14].

These deficiencies can be treated by intravenous fluid replacement or incorporation of phosphate into CRRT fluids. However, the addition of phosphate to CRRT fluids may alter systemic calcium and poses a risk of calcium precipitation [12, 15]. Furthermore, caution is required when using potassium-containing replacements, such as potassium chloride and potassium phosphate. Phosphate- and potassium-containing dialysis solutions are now also in use. Chua et al. and Broman et al. reported that a phosphate-containing dialysis solution reduced variability in the serum phosphate level during CRRT, and the incidence of hypophosphatemia [16, 17]. Our center has used both phosphate- and potassium-free dialysis solutions in patients undergoing CRRT; in July 2016, we started using potassium-containing dialysis solution, which was followed by the introduction of a phosphate- and potassium-containing solution. Three different protocols are followed when using these different dialysis solutions. This study was performed to compare the stability of serum phosphate and potassium levels among patients treated using these three dialysis solutions and protocols.

## Methods

### Study design and composition of study fluid

We studied a retrospective cohort of ICU patients with acute kidney injury (AKI) seen from January 2015 to December 2018 in Kangnam Sacred Heart Hospital. We used Hemosol-B0 (Gambro Lundia AB, Lund, Sweden) as a phosphate- and potassium-free dialysis solution; MultiBic (Fresenius Medical Care, Bad Hamburg, Germany), Prismasol 2 (Gambro Lundia AB), and Prismasol 4 (Gambro Lundia AB) were used as potassium-containing solutions, and Phoxilium (Gambro Lundia AB) as a phosphate- and potassium-containing solution. In our ICU, we follow three CRRT protocols. Patients were divided into three groups according to the dialysate and replacement solutions received, as follows: group 1 (January 1, 2015–June 30, 2016) received Hemosol B0 (no potassium or phosphate) as both dialysis and replacement fluids; group 2 (July 1, 2016–July 12, 2017) received Hemosol B0 and potassium-containing solution (MultiBic); and group 3 (July 13, 2017–December 30, 2018) received phosphate- and potassium-containing solution (Phoxilium), Hemosol B2, Prismasol 2, and Prismasol 4. Table 1 shows the dialysis fluid composition for each group; the treatment protocols are shown in Table 2. The dialysis solution used in each case was determined based on the patient’s phosphate and potassium levels. The ethic committee of Hallym University Kangnam Sacred Heart Hospital approved this study (IRB No 2019-05-027). We could not obtain the informed consent for the patients because we used de-identified and retrospective data. This issue also was confirmed by the hospital’s Institutional Review Board.

**Table 1:** Composition of dialysis fluid.

Composition (mmol/l)	Hemosol B0	MultiBic	Phoxilium	Prismasol 2	Prismasol 4
Sodium	140	140	140	140	140
Potassium	0	4	4	2	4
Calcium	1.75	1.5	1.25	1.75	1.75
Bicarbonate	32	35	30	32	32
Phosphorus	0	0	1.2	0	0
Chloride	109.5	113	116	111.5	113.5
Lactate	3	0	0	3	3
Magnesium	0.5	0.5	0.6	0.5	0.5
Glucose	0	5.5	0	6.1	6.1

**Table 2:** CRRT treatment protocols in intensive care unit.

Group	Criteria		Treatment		
	Serum K (mmol/L)	Serum P (mg/dL)	Dialysate Fluid	Pre-Replacement Fluid	Post-Replacement Fluid
1	K > 4.0		Hemosol B0	Hemosol B0	Hemosol B0
	K < 4.0		add 20meq KCL	add 20meq KCL	
2	K ≤ 4.5		Multibic 4K	Multibic 4K	Hemosol B0
	4.5 < K < 5.1		Multibic 4K	Hemosol B0	Hemosol B0
	K ≥ 5.1		Hemosol B0	Hemosol B0	Hemosol B0
3	K > 6.0	P ≥ 3.6	Hemosol B0	Hemosol B0	Hemosol B0
	4.5 < K ≤ 6.0	P ≥ 3.6	Prismasol 2	Prismasol 2	Prismasol 2
	4.5 < K ≤ 6.0	P ≤ 3.5	Phoxilium	Prismasol 2	Prismasol 2
	3.5 < K ≤ 4.5	P ≥ 3.6	Prismasol 4	Prismasol 2	Prismasol 2
	3.5 < K ≤ 4.5	P ≤ 3.5	Phoxilium	Prismasol 2	Prismasol 2
	K ≤ 3.5	P ≥ 3.6	Prismasol 4	Prismasol 2	Prismasol 2
	K ≤ 3.5	P ≤ 3.5	Phoxilium	Prismasol 4	Prismasol 2

### Patients and CRRT treatment

Patients admitted to the ICU with AKI who required CRRT were included in this study. Patients were excluded if they had chronic kidney disease or had received intermittent hemodialysis, if their CRRT treatment lasted < 24 hours, if they had cancer, or if they were under 18 years of age. Ultimately, we included 324 AKI patients who had been admitted to the ICU and received CCRT treatment. The Gambro Prismaflex CRRT machine, in continuous veno-venous hemodiafiltration (CVVHDF) mode, and a Hospal M100 filter (Hospal Industrie, Meyzieu, France) were used. Blood flow was set at 150 mL/min and a flow rate of dialysate and replacement fluid was set at 2,000 mL/h. Of the replacement fluids, 200 mL/h was post-filter and the rest was pre-filter. CRRT started without anticoagulants and if it needed set changes more than two times a day due to clotting, nafamostat was used as an anticoagulant. Fluid removal was set according to each patient’s condition and requirements. Intravenous phosphate and potassium were given when serum phosphate was < 2.6 mg/dL and serum potassium was < 3.5 mmol/L, or according to the general standard of care.

### Clinical parameters

The baseline patient characteristics recorded included age, gender, main cause of ICU admission, and CRRT treatment duration. Severity of illness was determined using the Acute Physiology and Chronic Health Evaluation II (APACHE II) score and the Glasgow Coma Scale (GCS). The mean blood pressure at ICU admission was also recorded. Blood samples were drawn from the arterial port of the venous catheter every 8 hours during CRRT treatment, to measure serum sodium, potassium, phosphate, ionized calcium, pH, and bicarbonate.

### Statistical analysis

Categorical variables are expressed as frequency (percentage) and were compared using the χ2 or Fisher’s probability test. Continuous variables are expressed as the mean (standard deviation) and were compared by analysis of variance (ANOVA). The rates of hypophosphatemia and hypokalemia of the three groups were evaluated using a negative binomial regression model including age, sex, BMI, APACHE II score, GCS, and serum baseline potassium or phosphate levels as covariates. The exposure variable in this model was CRRT duration. We applied the Bonferroni correction for multiple comparisons. Serum electrolyte values were analyzed longitudinally using a linear mixed model. The base model was adjusted for age, sex, APACHE II score, GCS, and main cause of ICU admission, and electrolyte values in each group were analyzed over time by using interaction term. In all analyses, p < 0.05 was taken to indicate statistical significance. Statistical analyses were performed using Stata/MP software (version 16.0; Stata Corp, College Station, TX).

## Results

The study population consisted of 324 patients undergoing CRRT, divided into group 1 (n = 105), group 2 (n = 78), and group 3 (n = 141). Age, use of vasopressor and mechanical ventilator, anticoagulant, main diagnosis leading to ICU admission and CRRT duration did not differ significantly among the three groups (Table 3). Patients in group 3 had significantly lower illness severity scores compared to those in groups 1 and 2 (both p < 0.001). 30-day mortality rates after ICU admission were not significantly different among the groups: group 1, 51.4%; group 2, 37.2%; group 3, 48.9%; p = 0.131).

**Table 3:** Baseline characteristics.

	Group 1	Group 2	Group 3
	N = 105	N = 78	N = 141
Age (year)	65.6 ± 16.9	65.4 ± 17.1	68.0 ± 13.5
Gender, Male	51 (48.6)*	52 (66.7)	95 (67.4)
BMI (kg/m^2^)	22.6 ± 4.0	22.7 ± 4.3	23.1 ± 4.2
Mean blood pressure (mmHg)	83.4 ± 20.9	82.6 ± 19.6	82.9 ± 16.9
Vasoactive drugs	88 (83.8)	66 (84.6)	128 (90.8)
Anticoagulant	104 (99.1)	76 (97.4)	140 (99.3)
CRRT treatment duration (day)	7.3 ± 6.5	6.5 ± 6.9	7.3 ± 8.9
Mechanical ventilation	82 (78.1)	54 (69.2)	104 (73.8)
APACHE2 score	17.6 ± 9.7*	13.9 ± 10.8**	10.5 ± 5.0
GCS score	8.0 ± 4.8	9.0 ± 4.9	8.5 ± 4.8
Contributing factors			
Sepsis	52 (49.5)	42 (53.9)	76 (53.9)
Hemodynamic instability without sepsis	52 (49.5)	33 (42.3)	60 (42.6)
Major surgery	1 (1.0)	3 (3.8)	5 (3.5)

* P<0.05 Group1 versus Group3

**P<0.05 Group2 versus Group3.

Data expressed as mean ± standard deviation and number (percent).

CRRT, continuous renal replacement therapy; APACHE II, Acute Physiology and Chronic Health Evaluation; GCS, Glasgow Coma Scale; BMI, body mass index.

Table 4 shows the incidence rate ratios (IRRs) of hypophosphatemia and hypokalemia during CRRT treatment. The IRRs of hypophosphatemia (1.14, 95% confidence interval (CI): 0.87 to 1.50) and hypokalemia (0.69, 95% CI: 0.39 to 1.20) between group 2 and group 1 were not statistically significant. However, group 3 had lower incidences of hypophosphatemia and hypokalemia compared to groups 2 and 1 when adjusted for age, sex, BMI, APACHE II score, GCS, and baseline serum phosphate or potassium level. The incidence rate of hypophosphatemia in group 3 was 55% lower compared to that in group 1 (IRR 0.45, 95% CI: 0.33 to 0.61) and 61% lower compared to that in group 2 (IRR 0.39, 95% CI: 0.29 to 0.53). Group 3 also had a 50% (IRR 0.50, 95% CI: 0.29 to 0.88) lower incidence rate of hypokalemia compared to group 1. However, the IRR of hypokalemia compared to group 2 was not significant (IRR 0.74, 95% CI: 0.41 to 1.33). Females had higher incidence rates of hypophosphatemia and hypokalemia compared to males after adjusting for other covariates. A higher GCS contributed to a higher incidence rate of hypokalemia after adjusting for other covariates. Serum baseline phosphate and potassium levels contributed to higher incidence rates of hypophosphatemia and hypokalemia. Regarding the presence of phosphate and potassium replacement in the three groups, 62 of 105 (59.1%) patients in group 1, 36 of 78 (46.2%) patients in group 2, and 16 of 141 (11.4%) patients in group 3 received one or more phosphate replacements (p < 0.001); 105 of 105 (100%) patients in group 1, 64 of 78 (82.1%) patients in groups 2, and 58 of 141 (41.1%) patients in group 3 were administered potassium replacement (p < 0.001). Hypoglycemia (< 70mg/dl) occurrence rates didn’t show significant difference among the groups (group 1, 10.5%; group 2, 6.4%; group 3, 11.4%; p-value = 0.487).

**Table 4:** Incidence rate of hypophosphatemia and hypokalemia.

Hypophosphatemia			Hypokalemia		
	IRR (95% CI)	p-value		IRR (95% CI)	p-value
Group 2 vs. 1	1.14 (0.87–1.50)	0.698	Group 2 vs. 1	0.69 (0.39–1.20)	0.301
3 vs. 1	0.45 (0.33–0.61)	<0.001	3 vs. 1	0.50 (0.29–0.88)	0.010
3 vs. 2	0.39 (0.29–0.53)	<0.001	3 vs. 2	0.74 (0.41–1.33)	0.658
Age	1.00 (0.99–1.0)	0.550	Age	0.98 (0.97–0.99)	0.004
BMI	0.97 (0.95–0.99)	0.032	BMI	0.97 (0.92–1.01)	0.172
Female (vs. Male)	1.20 (0.99–1.45)	0.065	Female (vs. Male)	1.49(1.03–2.17)	0.034
APACHEII	1.0 (0.99–1.01)	0.772	APACHEII	0.99 (0.96–1.02)	0.451
GCS	1.0 (0.98–1.02)	0.792	GCS	1.08 (1.03–1.13)	<0.001
Serum P_baseline	0.92 (0.88–0.96)	<0.001	Serum K_baseline	0.53 (0.43–0.66)	<0.001

IRR, incidence rate ratio; CI, confidence interval; RRT, continuous renal replacement therapy; APACHE II, Acute Physiology and Chronic Health Evaluation; GCS, Glasgow Coma Scale Hypophosphatemia is defined as serum phosphate was < 2.6 mg/dL, and hypokalemia as serum potassium was < 3.5 mmol/L.

**Fig 1:**
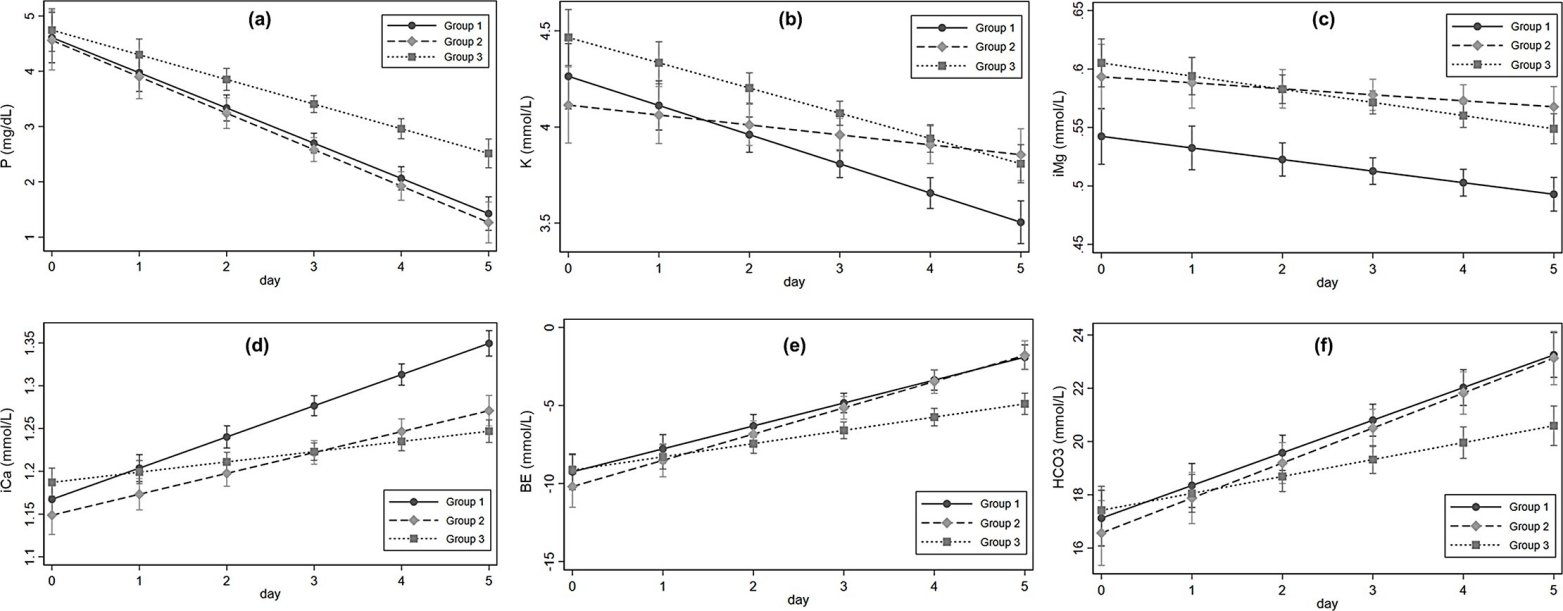
Adjusted marginal values (95% CI) of serum electrolyte during CRRT.

Fig 1 shows the adjusted predictive marginal values and 95% CIs of serum electrolytes for mixed linear regression analysis. Fig 1A shows the serum phosphate levels over time. The baseline serum phosphate levels were not different among the three groups (group 2 vs. 1: ß = −0.08, 95% CI: −0.79 to 0.62, p = 0.821; group 3 vs. 1: ß = 0.12, 95% CI: −0.48 to 0.73, p = 0.695). Regarding the extent of the change in serum phosphate level over time, the slope in group 2 was not different from that in group 1 (ß = −0.22, 95% CI: −0.23 to 0.18, p = 0.836). However, group 3 had a less negative slope (ß = 0.19, 95% CI: 0.02 to 0.37, p = 0.032) than group 1. Serum potassium levels in the three groups decreased over time (Fig 1B). The decrease was significantly lesser in group 2 compared to group 1 (ß = 0.10, 95% CI: 0.03 to 0.17, p = 0.008), but the slope was not different between groups 3 and 1 (ß = 0.02, 95% CI: −0.04 to 0.08, p = 0.526). The slopes of the baseline ionized magnesium levels were not different between groups 2 and 1 (ß = 0.005, 95% CI: −0.005 to 0.01, p = 0.321) or groups 3 and 1 (ß = −0.001, 95% CI: −0.009 to −0.007, p = 0.742) (Fig 1C). The ionized calcium levels increased and decreased in groups 2 and 3, respectively, relative to in group 1 over time (group 2 vs. 1: ß = −0.01, 95% CI: −0.02 to −0.004, p = 0.002; group 3 vs. 1: ß = −0.02, 95% CI: −0.03 to −0.02, p < 0.001) (Fig 1D). However, none of the patients received intravenous calcium. Base excess (BE) and bicarbonate levels also increased and changes over time were not different between groups 1 and 2 (Fig 1E and 1F). The levels remained lower in group 3 compared to groups 1 and 2 (BE: ß = −0.62, 95% CI: −1.0 to −0.24, p = 0.001; bicarbonate: ß = −0.59, 95% CI: −0.97 to −0.21, p = 0.003). However, in group 3 the bicarbonate level remained > 20 mmol/L and acid-base disturbances were well controlled.

## Discussion

In this study, the decline in serum phosphate during CRRT was effectively prevented by the use of phosphate-containing dialysis solution, despite the decreased use of intravenous phosphate. The rate of hypophosphatemia was higher in patients receiving phosphate-free dialysis solution. Serum potassium levels during CRRT remained steady in patients receiving potassium-containing dialysis solution and the rate of hypokalemia was lower in group 3 than in group 1. The rate of hypokalemia was not different between groups 2 and 3. However, the potassium level during CRRT remained > 3.5 mmol/L in group 3 and the rate of intravenous potassium was significantly higher in group 2 compared to group 3. The CRRT protocol differed between groups 2 and 3, which may explain the difference in intravenous potassium use.

Hypophosphatemia promotes mortality and is associated with a variety of complications in critically ill patients [8, 18]. The large amount of ultrafiltration in CVVHDF leads to convective transfer of electrolytes across the membrane and causes electrolyte imbalances. Hypophosphatemia is the most frequently observed electrolyte abnormality, occurring in about 20–60% of patients [5, 19]. Hypokalemia can also develop during CRRT, although its incidence is lower than that of hypophosphatemia. Several recent studies have investigated the usefulness of phosphate- and potassium-containing dialysis solution [16, 17, 20, 21]. Broman et al. reported that phosphate-containing replacement and dialysis solutions reduced variability in serum phosphate levels during CRRT and the incidence of hypophosphatemia [17]. In a crossover study, Noemie et al. reported that phosphate- and potassium-containing solution prevented hypophosphatemia and hypokalemia during CVVHDF [20].

Here, we confirmed that hypophosphatemia and hypokalemia during CRRT can be prevented using phosphate- and potassium-containing solution. In addition, the rate of intravenous replacement was reduced using this solution. We implemented management protocols according to the levels of phosphate and potassium. To control electrolyte imbalance during CRRT, the protocol followed for group 3 was advantageous in terms of the hypokalemia, hypophosphatemia and intravenous replacement rates. These results showed that the use of different solutions and protocols according to the electrolyte levels of individual cases may effectively prevent electrolyte variability and reduce the requirement for intravenous replacement.

The use of phosphate- and potassium-containing dialysis solution did lead to other electrolyte imbalances or the requirement for intravenous replacement. The extent of the hypocalcemia induced by Phoxilium and Hemosol-B0 has been compared in previous studies [17]. Phoxilium has a lower calcium content than Hemosol-B0. In this study, ionized calcium levels remained lower over time in group 3 compared to group 1. However, the lowered serum calcium levels were still within the physiological range and none of the patients received intravenous calcium. In addition, there was no disturbance in the magnesium level. Important concerns associated with using phosphate- and potassium-containing dialysis solution include whether these fluids can cause phosphate and potassium overload. However, in our study, the incidence rates of hyperkalemia and hyperphosphatemia did not differ among the three groups after 24 hours of CRRT. The phosphate-containing dialysis solution may have caused a slight decrease in serum bicarbonate and BE levels, because this solution contains lower amounts of bicarbonate compared to conventional solutions. Furthermore, the hyperphosphatemia associated with the phosphate-containing solution may have led to metabolic acidosis, given the weakly acidic properties of the solution. The results of this study were consistent with previous studies regarding serum bicarbonate and BE levels [16, 17, 20]. However, most cases of acid-base disturbance were well controlled using our protocols.

This study had several limitations. First, we did not have any data on the actual CRRT doses delivered. However, our center has previously established a standard CRRT dose, which is not high enough to cause high dialysis intensity. Second, this study has inherent weakness like all other studies using a retrospective design due to use of past medical records. Thus, we couldn’t evaluate exact causes of AKI. There might remain potential confounding factors such as CRRT indications and ICU admission in sequential three periods with different APACHEII scores. However, the APACHEII score indicating illness severity was not an associating factor of hypophosphatemia and hypokalemia. On the CRRT start day, there were no significant differences among the three groups in serum phosphate or potassium levels, nor in the main diagnosis leading to ICU admission. Third, we didn’t apply individualized CRRT treatments to the patients. Instead, we used the same blood flow and CRRT dose to all patients. CRRT dose (kg/ml/h), which is defined as dialysate and replacement fluid flow rate divided by weight, might be higher in women than men. It could explain the higher rates of hypophosphatemia and hypokalemia in women.

## Conclusion

We confirmed that the use of phosphate- and potassium-containing solution effectively prevents hypophosphatemia and hypokalemia, and attenuates changes in serum phosphate and potassium levels during CRRT. It also avoids the need for intravenous replacement. The protocol used in this study may significantly reduce the workload of nurses, and the number of errors occurring during intravenous electrolyte replacement.

## Supporting information

S1 Data (XLS)
